# Menke-Hennekam syndrome 1: A Case Report

**DOI:** 10.1192/j.eurpsy.2022.1109

**Published:** 2022-09-01

**Authors:** M. Trusmei, M. Budisteanu

**Affiliations:** University Titu Maiorescu, Faculty Of Medicine, bucharest, Romania

**Keywords:** developmentaldelay, Menke-Hennekamsyndrome, dysmorphicfeatures, autisticbehaviour

## Abstract

**Introduction:**

Menke-Hennekam syndrome (MHS) is a relatively new genetic condition characterized by intellectual disabilities, autistic behavior, auditory defects, recurrent upper respiratory tract infections, microcephaly and short stature. Facial characteristics include short palpebral fissures, telecanthus, depressed nasal bridge, short nose, anteverted nares, short columella, and long philtrum. The genetic defect is represented by missense variants of *CREBBP* gene, located on exons 30 or 31. There are only around 30 cases reported by now.

**Objectives:**

The aim of the paper is to report a new case of MHS.

**Methods:**

The case is a 3-year-old boy admitted in our department for developmental delay. The clinical examination revealed dysmorphic features; severe speech delay, mild intellectual disability, autistic behaviour.The patient had a personal history of recurrent respiratory infections, visual defect and bilateral sensorineural hearing loss. Other investigations included EEG, abdominal echography, and cerebral MRI all were normal. The genetic studies included array CGH and WES.

**Results:**

The array CGH was normal. WES identified a pathogenic heterozygote variant c.5600G>A in the exon 31 of CREBBP gene, confirming MHS.

**Conclusions:**

Overall, the features of our patient are consistent with those reported in the previous reports, including developmental and speech delay, autistic behavior, dysmorphic features, recurrent upper way infections, sensorineural hearing loss, and visual defects. Other common features, such as growth delay and microcephaly were not present in our patient. Our case contributes to the clinical characterisation of the new syndrome. Funding: The research leading to these results has received funding from the EEA Grant 2014-2021, under the project contract No 6/2019.

**Disclosure:**

No significant relationships.

